# Supplementary figures and images for: Whole Genome Resequencing Reveals Selection Signals Related to Wool Color in Sheep
Source: Animals (Basel). 2023 Oct 19;13(20):3265. doi: 10.3390/ani13203265 (PMC10603731; doi:10.3390/ani13203265)

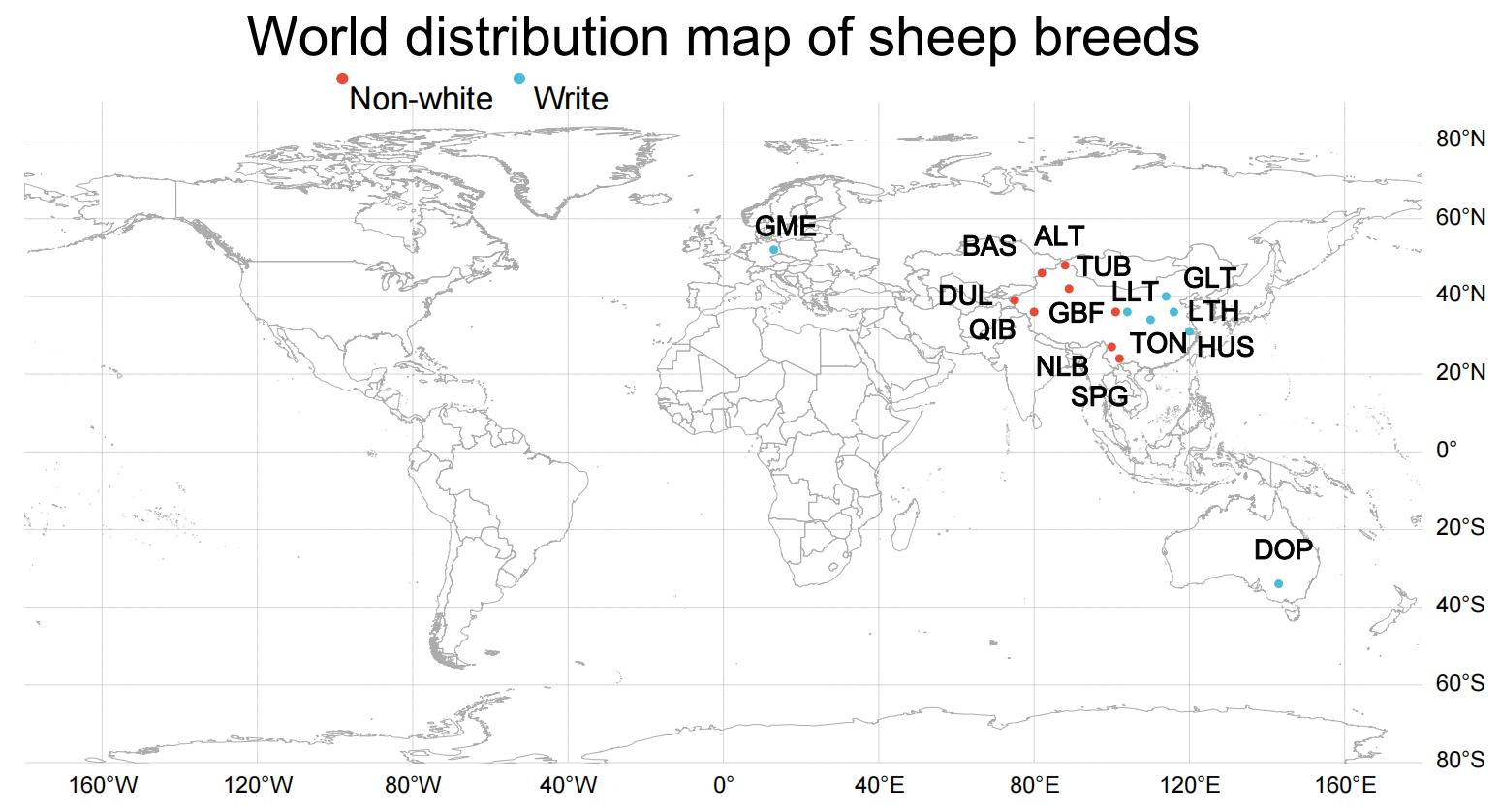

Supplement: Supplementary file 1 [file animals-13-03265-s001.zip › Figure S1.png]

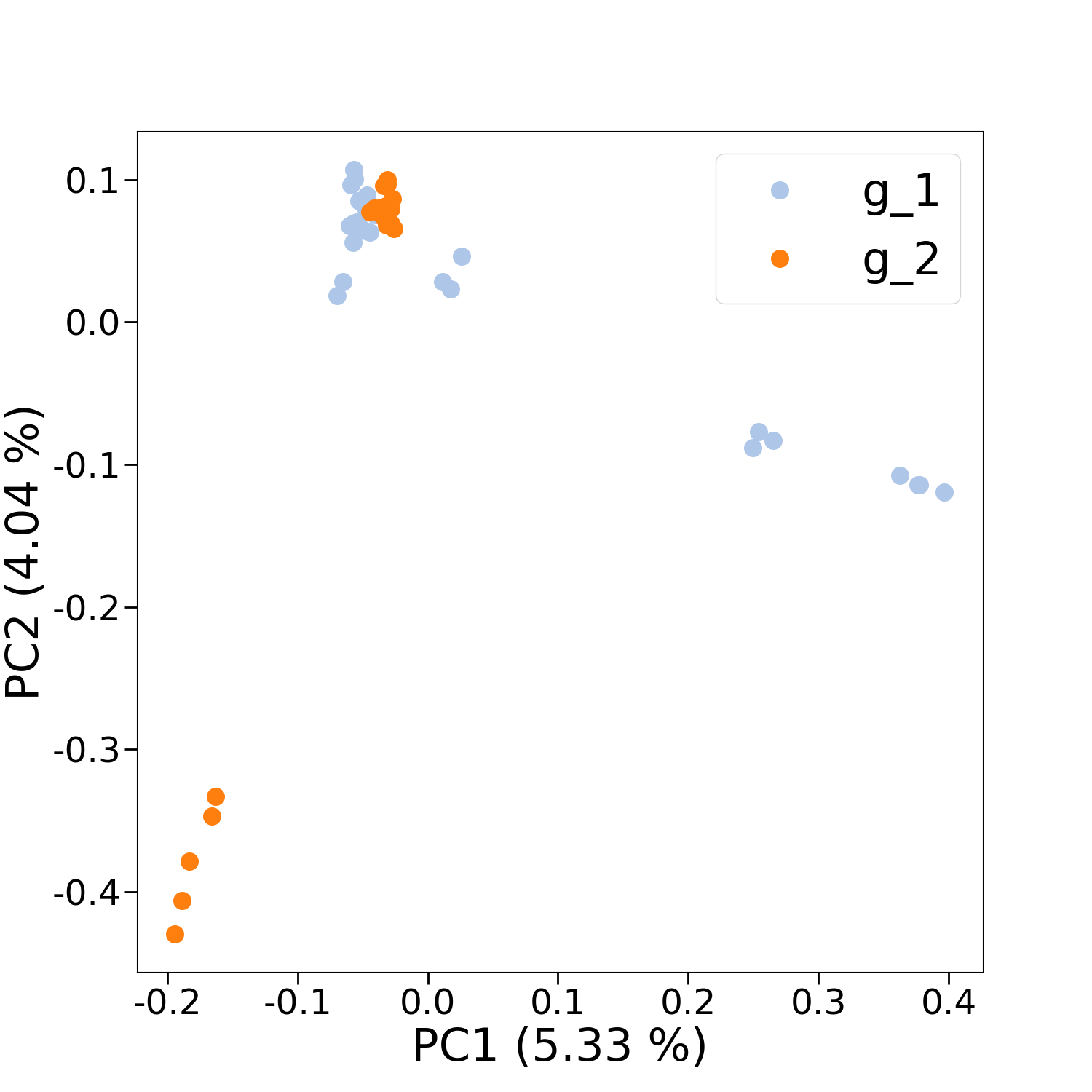

Supplement: Supplementary file 1 [file animals-13-03265-s001.zip › Figure S2.png]

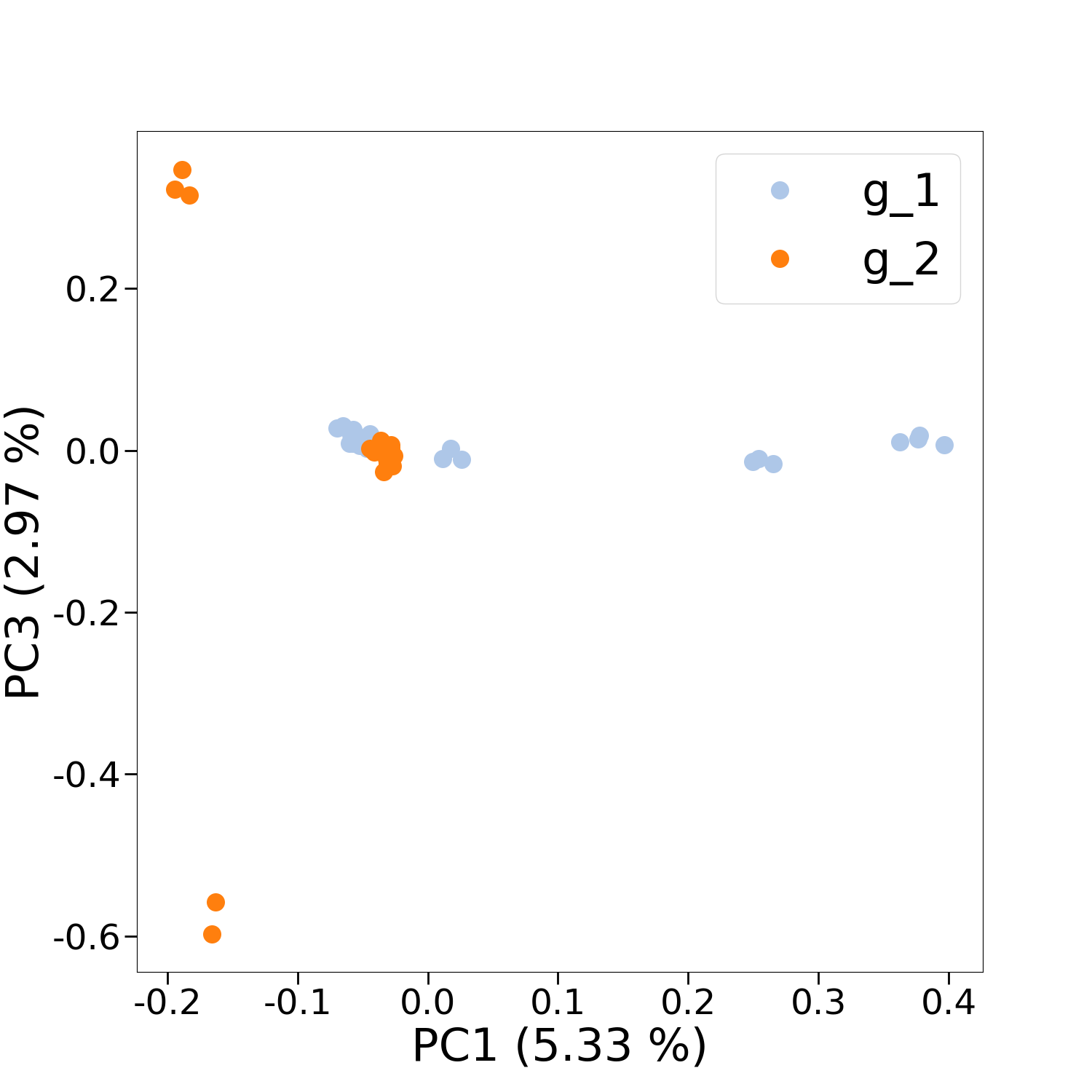

Supplement: Supplementary file 1 [file animals-13-03265-s001.zip › Figure S3.png]

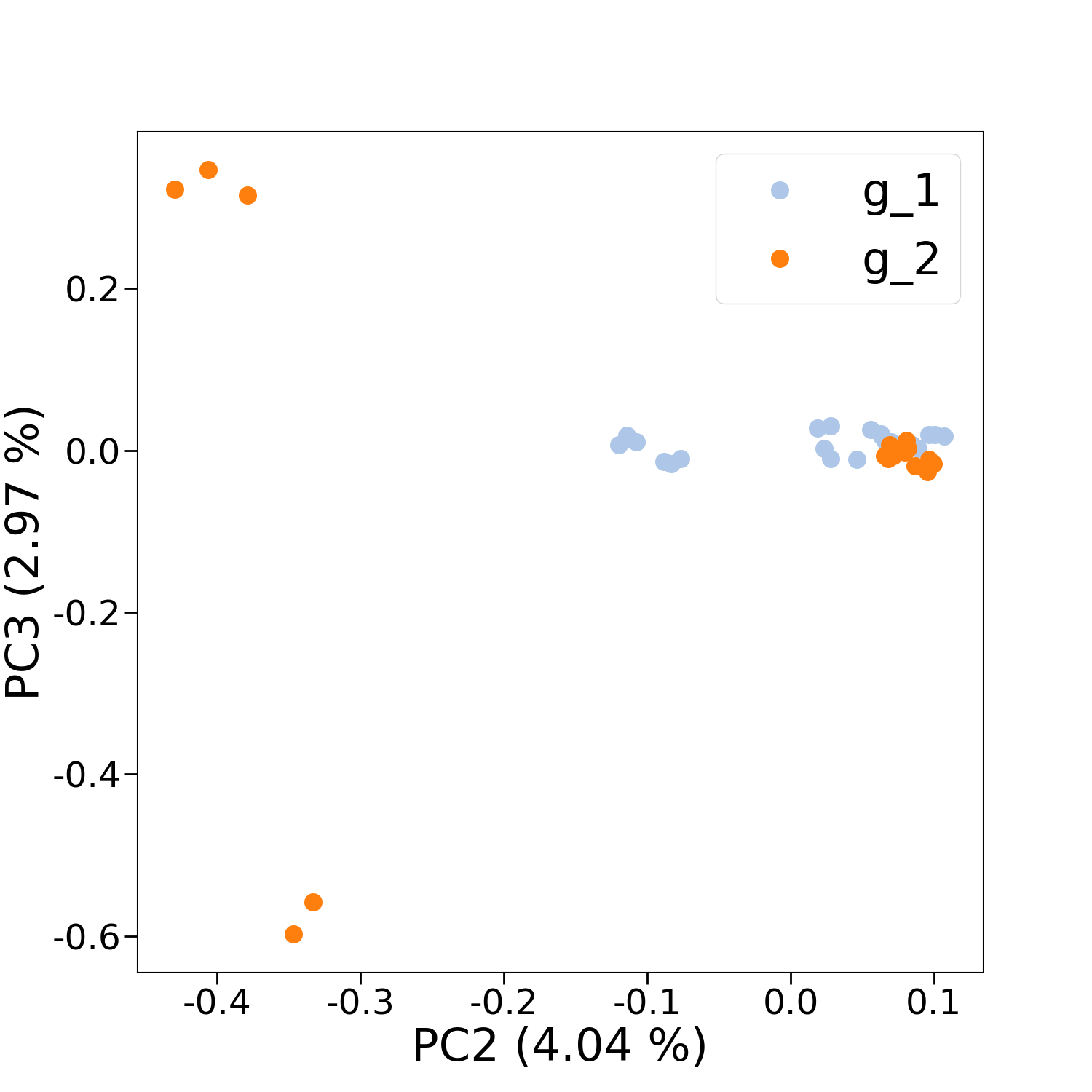

Supplement: Supplementary file 1 [file animals-13-03265-s001.zip › Figure S4.png]
